# Human Mesenchymal Stem Cell Secretome from Bone Marrow or Adipose-Derived Tissue Sources for Treatment of Hypoxia-Induced Pulmonary Epithelial Injury

**DOI:** 10.3390/ijms19102996

**Published:** 2018-09-30

**Authors:** Nala Shologu, Michael Scully, John G. Laffey, Daniel O’Toole

**Affiliations:** 1 The Discipline of Anesthesia and Lung Biology Group, National University of Ireland Galway, H91 TK33 Galway, Ireland; john.laffey@nuigalway.ie (J.G.L.); daniel.otoole@nuigalway.ie (D.O.); 2 Critical Care Medicine and Intensive Care, University Hospital Galway, H91 YR71 Galway, Ireland; michael.scully@nuigalway.ie

**Keywords:** mesenchymal stem cells, MSC therapy, MSC conditioned medium, hypoxia, hypoxia-reoxygenation, lung ischemia-reperfusion, alveolar epithelial cells, heat shock proteins, glucose-regulated proteins, acute lung injury

## Abstract

Alveolar epithelial dysfunction induced by hypoxic stress plays a significant role in the pathological process of lung ischemia-reperfusion injury (IRI). Mesenchymal stem cell (MSC) therapies have demonstrated efficacy in exerting protective immunomodulatory effects, thereby reducing airway inflammation in several pulmonary diseases. Aim: This study assesses the protective effects of MSC secretome from different cell sources, human bone marrow (BMSC) and adipose tissue (ADSC), in attenuating hypoxia-induced cellular stress and inflammation in pulmonary epithelial cells. Methods: Pulmonary epithelial cells, primary rat alveolar epithelial cells (AEC) and A549 cell line were pre-treated with BMSC, or ADSC conditioned medium (CM) and subjected to hypoxia for 24 h. Results: Both MSC-CM improved cell viability, reduced secretion of pro-inflammatory mediators and enhanced IL-10 anti-inflammatory cytokine production in hypoxic injured primary rat AECs. ADSC-CM reduced hypoxic cellular injury by mechanisms which include: inhibition of p38 MAPK phosphorylation and nuclear translocation of subunits in primary AECs. Both MSC-CM enhanced translocation of Bcl-2 to the nucleus, expression of cytoprotective glucose-regulated proteins (GRP) and restored matrix metalloproteinases (MMP) function, thereby promoting repair and cellular homeostasis, whereas inhibition of GRP chaperones was detrimental to cell survival. Conclusions: Elucidation of the protective mechanisms exerted by the MSC secretome is an essential step for maximizing the therapeutic effects, in addition to developing therapeutic targets-specific strategies for various pulmonary syndromes.

## 1. Introduction

Mesenchymal stem cells (MSC), are increasingly being used in pulmonary research for preclinical and clinical applications [1]. There are several sources for the isolation of MSCs, including but are not limited to adipose tissue, bone marrow, umbilical cord, infrapatellar fat pad, synovial fluid, foetal tissues, periosteum and amniotic fluid [2,3]. Depending on environmental stimuli, these cells have the ability to develop into adipose tissues, bone, cartilage, vessels, muscle, tendon and marrow [4]. Nevertheless, though the current understanding on MSC mechanisms of action and biology has paved the way to preclinical and clinical trials, further studies are needed to explain and improve the efficacy of the current MSC therapies, particularly for pulmonary diseases. Heat shock proteins (HSP) are known to be expressed in various cell types and are enhanced by several cellular stressors such as free radicals, thermal stress and hypoxia [5]. Studies have shown that preconditioning MSCs with HSPs protects against hypoxia-induced apoptosis [6]. Stress-inducible intracellular chaperones, glucose-regulated proteins (GRP), GRP75, GRP78 and GRP94, belong to the HSP family. In addition to their traditional chaperone roles of protein folding and assembly, these GRPs also assume novel functions that control signalling, proliferation, invasion, apoptosis, inflammation and immunity [7]. MSC therapy may be a feasible approach to elevate GRP expression safely and efficiently as a strategy to protect against acute lung injury (ALI).

Lung ischemia-reperfusion injury (IRI) is a clinical complication that occurs after surgical procedures such as cardiopulmonary bypass or lung transplantation and remains a significant cause of morbidity and mortality [8]. Lung IRI is one of the leading causes of acute and long-term pulmonary complications, such as development of primary graft dysfunction (PGD) and significantly impacts patient survival after lung transplantation [9]. During ischemia, oxygen deprivation directly generates oxidative tissue damage and hypoxia, followed by reperfusion which results in the upregulation of inflammatory mediators, oedema and pulmonary infiltrates [10]. This ischemic injury is defined as an abrupt and extended disruption of arterial blood flow with instantaneous deprivation of oxygen and nutrients to the cells resulting in the build-up of cellular metabolic by-products [11]. In vivo studies of the mechanisms of hypoxic responses have been widely assessed, where vascular occlusion has been used to induce hypoxic-ischemic conditions [12] and lowered oxygen tension has been used for mimicking the reduced oxygen supply for in vitro ischemic models. Hypoxia/re-oxygenation has been employed by exposing lung cell culture systems to acute hypoxia followed by re-oxygenation under normoxic conditions [13], while other investigators have used non-hypoxic in vitro systems of cold ischemia using 4 °C preservation solution prior to re-oxygenation with 37 °C culture media [14,15,16]. The resulting decrease in the rate of oxygen supply decreases the cellular partial pressure of oxygen (PO_2_) and accordingly induces inflammation, further contributing to injurious immune responses [17]. Clinically and cellularly, hypoxia and inflammation are interconnected mechanisms down to the molecular level, as inflammation is a significant cause of hypoxia in addition to being caused by hypoxia [18].

Given that the typical environment for lung ischemia is sterile, both in vivo and in vitro microenvironments share several phenotypic similarities with the mechanism of the consequential hypoxic responses in relation to the activation of a host immune response [19]. Moreover, this study focuses on the protective effects of the MSC secretome on pulmonary epithelial cells injured by hypoxia. The mechanism by which the MSC secretome exerts its protective effects is hypothesized to be through the release of paracrine factors that regulate cellular stress signalling pathways and modulate the inflammatory response.

## 2. Results

### 2.1. Anti-Apoptotic Effects of MSC Secretome

**Figure 1:**
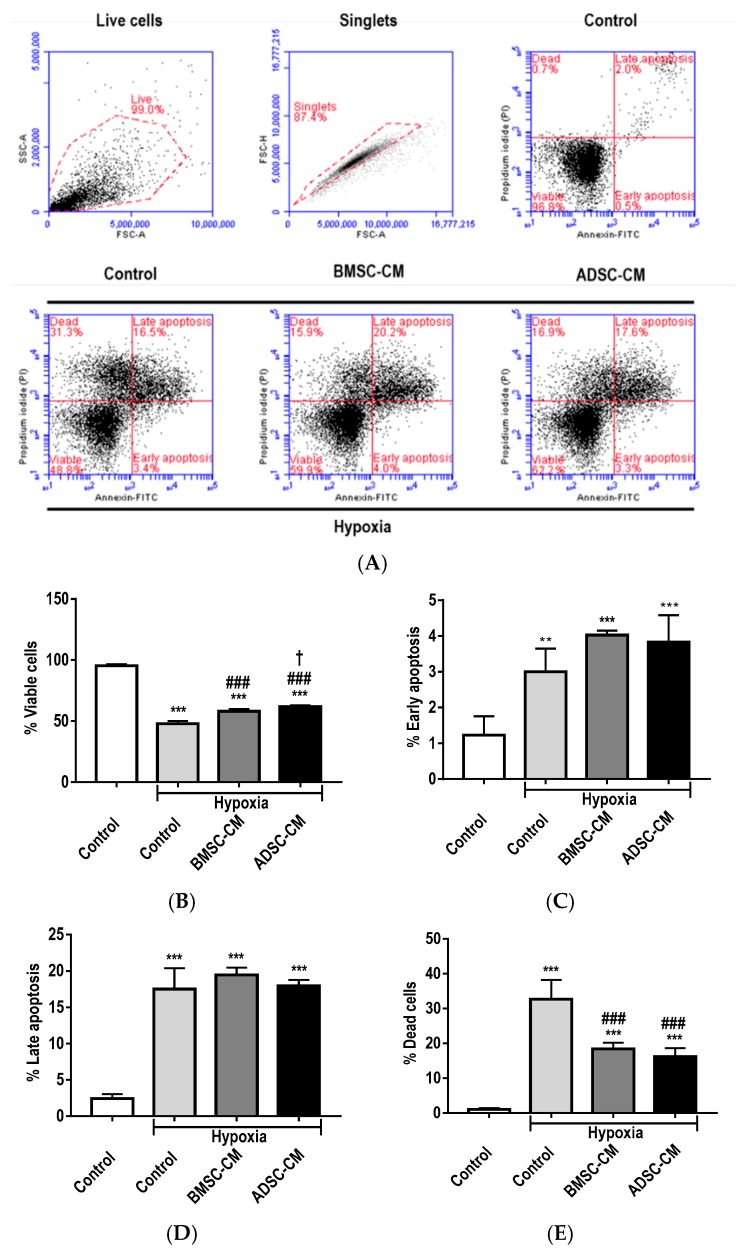
(**A**) Flow cytometry analysis of primary rat AECs treated with human MSC-CM during hypoxic (1.5% O_2_) exposure for 24 h. The percentage of cells in various apoptotic stages, (**B**) viable cells, (**C**) early apoptosis, (**D**) late apoptosis and (**E**) dead cells were collected from 10,000 single-cell events. Data presented as mean  ±  SD; *n* = 3 (* *p*  <  0.05, ** *p*  <  0.01, *** *p*  <  0.001 vs. normoxia control, # *p*  <  0.05 and ### *p*  <  0.001 vs. hypoxia control and † *p*  <  0.05 vs. BMSC-CM). The resulting data were statistically analysed using one-way ANOVA and Tukey’s multiple comparison test.

Flow cytometry assessment (Figure 1A) of primary rat alveolar epithelial cells (AEC) showed that hypoxic exposure significantly induced apoptosis. The percentage of viable cells was also significantly reduced (*p* < 0.001) by hypoxic exposure (Figure 1B). Pre-treatment of cells with both MSC-CM was significantly protective (*p* < 0.001) preserving cell viability compared to hypoxic control, with ADSC-CM being significantly superior (*p* < 0.05) to BMSC-CM. Although there was a small percentage of cells that were observed to be in early apoptosis, hypoxic exposure significantly increased (*p* < 0.01) the level of early apoptotic cells. Moreover, a further increase (*p* < 0.001) of early apoptotic cells was observed with MSC-CM pre-treatment (Figure 1C), which may be attributed to the MSC-CM delaying the cells entering late apoptosis. A similar percentage of primary AECs were observed in late apoptosis in both hypoxia control and MSC-CM pre-treated groups, displaying a similar percentage (*p* < 0.001) of late apoptotic cells (Figure 1D). There was a significantly higher (*p* < 0.001) percentage of dead cells observed with hypoxic treatment, however, pre-treatment with MSC-CM significantly (*p* < 0.001) attenuated this effect (Figure 1E). Moreover, pre-treatment with MSC-CM was effective at protecting cells against hypoxia-induced apoptosis.

### 2.2. Protective Effects of MSC Secretome

**Figure 2:**
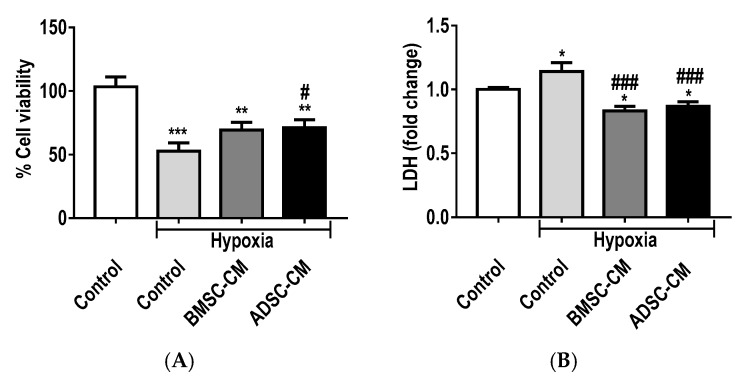
(**A**) Cell viability and (**B**) LDH release of primary rat AECs treated with human MSC-CM during hypoxic (1.5% O_2_) exposure for 24 h. The viability and LDH release of primary AECs were measured via MTT and LDH assays, respectively. Data presented as mean  ±  SD; *n* = 3 (* *p*  <  0.05, ** *p*  <  0.01, *** *p*  <  0.001 vs. normoxia control, # *p*  <  0.05 and ### *p*  <  0.001 vs. hypoxia control). The resulting data were statistically analysed using one-way ANOVA and Tukey’s multiple comparison test.

Cell viability by a measure of metabolic activity using [3-(4,5-dimethylthiazol-2-yl)-2,5-diphenyltetrazolium bromide] (MTT) assay (Figure 2A) revealed a significant decrease in the viability of cells exposed to hypoxia. While an increase is observed in the cell viability of MSC-CM treated primary rat AECs during hypoxic exposure (1.5% O_2_), a significant (*p* < 0.05) difference was observed in ADSC-CM treated cells compared to hypoxic control. Lactate dehydrogenase (LDH) assay (Figure 2B) demonstrated that both bone marrow-derived CM (BMSC-CM) and adipose-derived stem cell CM (ADSC-CM) treated groups were effective in significantly (*p* < 0.05) reducing LDH release compared to normoxic control. A further reduction (*p* < 0.001) of LDH release was observed compared to hypoxic control, whereas hypoxia control significantly increased (*p* < 0.05) LDH. Similar trends were observed with A549 (Figures S1 and S2) under more severe hypoxic exposure (0.5% O_2_). Treatment with both BMSC-CM and ADSC-CM proved to be cytoprotective, with preservation of cell viability and significant reduction of LDH (*p* < 0.01) compared to hypoxic control.

### 2.3. Anti-Inflammatory Effects of MSC Secretome

**Figure 3:**
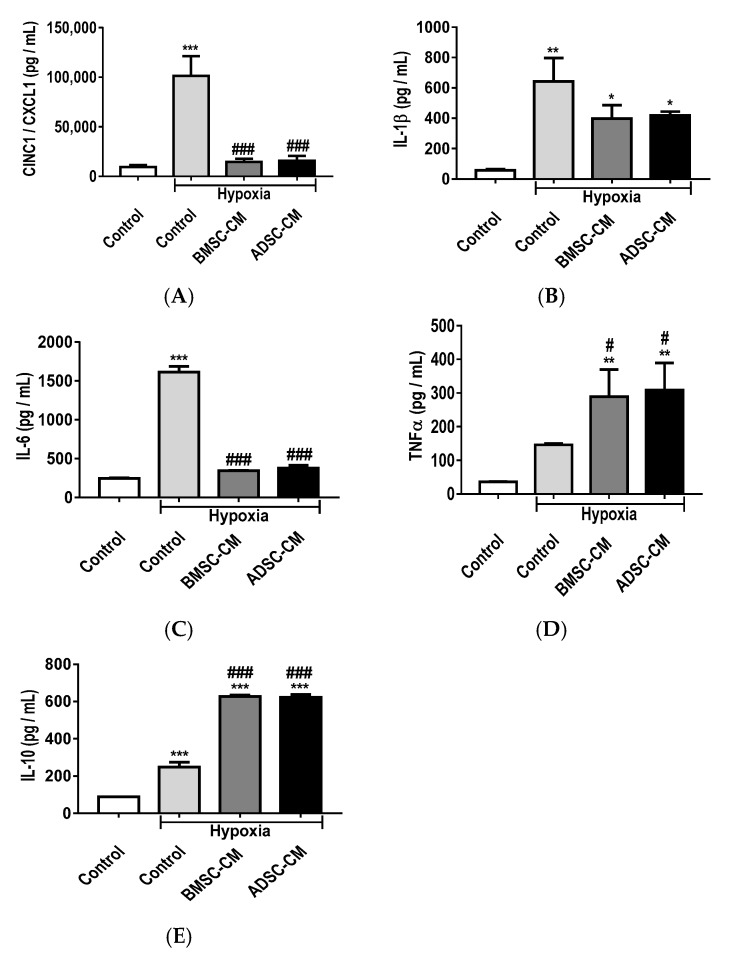
The release of pro-inflammatory; (**A**) CINC-1/CXCL-1, (**B**) IL-1β, (**C**) IL-6, (**D**) TNF-α and anti-inflammatory cytokines; (**E**) IL-10 from primary rat AECs pre-treated with human BMSC/ADSC-CM during hypoxic (1.5% O_2_) exposure for 24 h. ELISAs were used to measure secreted inflammatory cytokines in the supernatant of primary AECs. Data presented as mean  ±  SD; *n* = 3 (* *p*  <  0.05, ** *p*  <  0.01, *** *p*  <  0.001 vs. normoxia control; and # *p*  <  0.05. ### *p*  <  0.001 vs. hypoxia control). The resulting data were statistically analysed using one-way ANOVA and Tukey’s multiple comparison test.

Enzyme-linked immunosorbent assay (ELISA) has shown that hypoxia exposure significantly (*p* < 0.001) increased the release of an inflammatory cytokine, cytokine-induced neutrophil chemoattractant 1 (CINC-1), also known as chemokine (C-X-C motif) ligand-1 (CXCL-1), compared to normoxic control, in primary rat AECs (Figure 3A). BMSC-CM and ADSC-CM treated groups showed significantly (*p* < 0.001) reduced release of CINC-1/CXCL-1, restoring to levels similar to that of normoxic conditions. Hypoxia treatment also significantly (*p* < 0.01) increased the production of interleukin-1 beta (IL-1β) (Figure 3B) compared to normoxic control, demonstrating enhanced inflammation in primary AECs. The presence of BMSC-CM and ADSC-CM significantly (*p* < 0.05) reduced the release of IL-1β compared to hypoxia treatment, displaying the immunomodulatory effects of MSC secretome. Likewise, interleukin-6 (IL-6) release (Figure 3C) was significantly (*p* < 0.001) increased in hypoxia as compared to normoxic control. The presence of BMSC-CM and ADSC-CM significantly (*p* < 0.001) reduced pro-inflammatory cytokine production induced by hypoxia and no significant differences were observed compared to normoxic control. While the pro-inflammatory cytokine tumour necrosis factor alpha (TNF-α) (Figure 3D) was significantly (*p* < 0.01) increased in BMSC-CM and ADSC-CM treated groups compared to normoxic control, these effects were further significantly (*p* < 0.05) enhanced compared to hypoxia. Anti-inflammatory cytokine interleukin-10 (IL-10) (Figure 3E) production was significantly (*p* < 0.001) increased under hypoxic treatment compared to control and a further significant (*p* < 0.001) enhancement was observed in BMSC-CM and ADSC-CM treated groups compared to hypoxic control, demonstrating the protective effects of MSC secretome.

### 2.4. MSC Secretome Restores MMP Function during Cellular Injury

**Figure 4:**
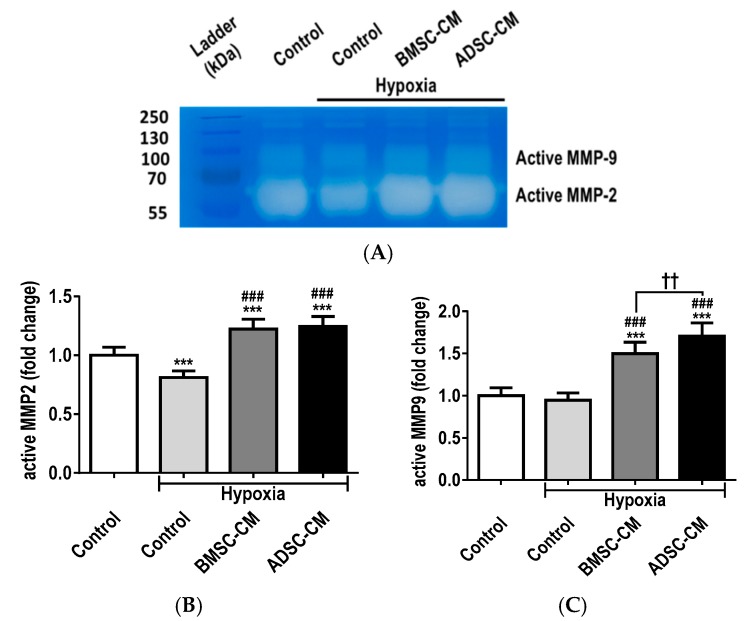
(**A**) Gelatin zymography for the detection of MMPs, (**B**) active MMP-2 and (**C**) MMP-9 protein secretion by primary rat AECs treated with human MSC-CM during hypoxic (1.5% O_2_) exposure for 24 h to assess injury index. The activities of active MMP-2 and MM-9 were quantified using densitometry. Data presented as mean  ±  SD; *n* = 3 (*** *p*  <  0.001 vs. normoxia control; ### *p*  <  0.001 vs. hypoxia control and †† *p*  <  0.01 vs. BMSC-CM). The resulting data were statistically analysed using one-way ANOVA and Tukey’s multiple comparison test.

Gelatin zymography (Figure 4A) demonstrated that the presence of MSC-CM had restorative capabilities, at least in part, because of matrix metalloproteinases (MMP), namely MMP-2 and MMP-9, which intimately regulate activities of soluble factors during injury and repair. Hypoxia significantly (*p* < 0.001) reduced MMP-2 secretion, thereby demonstrating these detrimental effects as an index of cellular injury and disruption of homeostasis. BMSC-CM and ADSC-CM significantly (*p* < 0.001) increased MMP-2 production compared to normoxic control, with further enhancement compared to hypoxia (Figure 4B). Similar trends were also observed with MMP-9 (*p* < 0.001) expression. However, ADSC-CM was observed to be superior to the BMSC secretome (Figure 4C) Thus, the restoration of activated MMP-2 and MMP-9 by the MSC-CM is likely critical for recovery to physiological homeostasis.

### 2.5. Cytoprotective Effects of MSC Secretome

**Figure 5:**
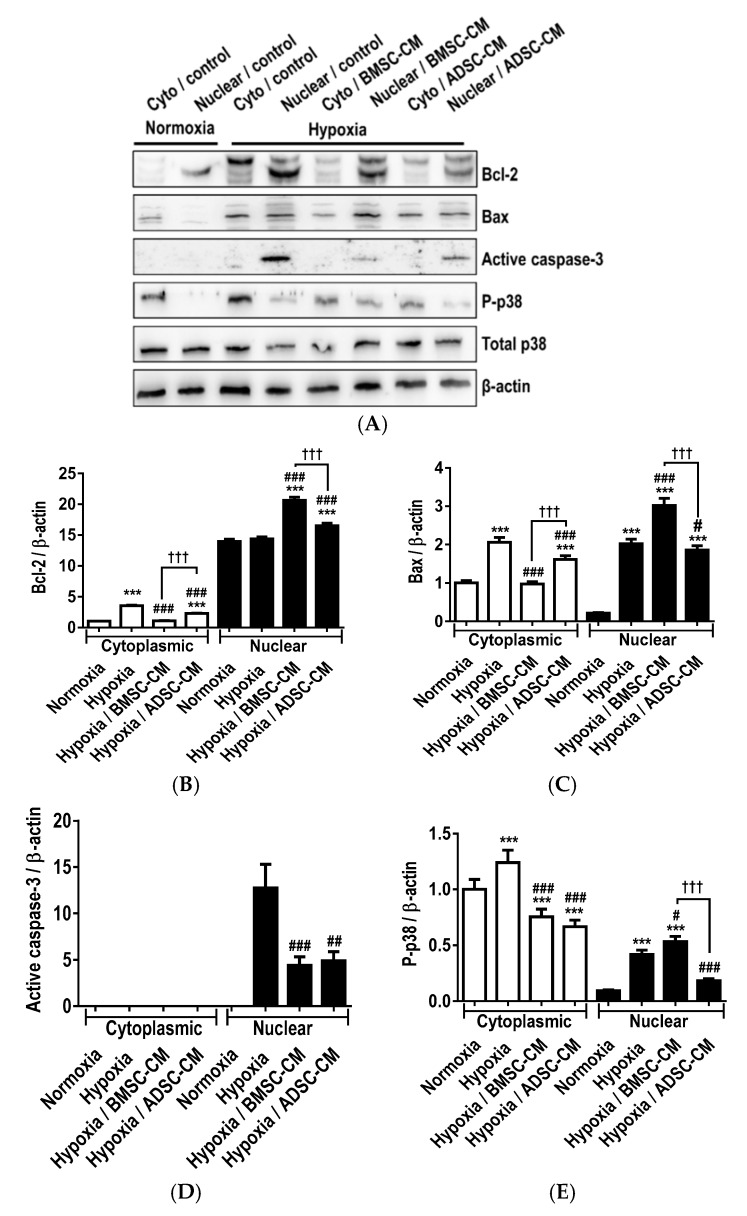
(**A**) Western blot analysis exploring modulatory effects of human MSC-CM on the activation of cellular stress signalling mediators in primary rat AECs exposed to hypoxia (1.5% O_2_) for 24 h Cytoplasmic and nuclear accumulation of (**B**) Bcl-2, (**C**) Bax, (**D**) activate caspase-3 and (**E**) phosphorylated p38 were quantitated by densitometry and normalized to β-actin. Data presented as mean  ±  SD; *n* = 3 (*** *p*  <  0.001 vs. normoxia control; # *p*  <  0.05. ## *p*  <  0.01, ### *p*  <  0.001 vs. hypoxia control and ††† *p*  <  0.001 vs. hypoxia/BMSC-CM). The resulting data were statistically analysed using one-way ANOVA and Tukey’s multiple comparison test.

Primary rat AECs pre-treated with MSC-CM and subjected to hypoxia (1.5% O_2_) exposure were analysed by Western blot (Figure 5A). Modulation of protein expression and activation of Bcl-2, Bax active caspase-3 and phosphorylated p38 mitogen-activated protein kinase (MAPK) were assessed to confirm induction of anti-apoptotic, cytoprotective and cellular stress signalling pathways. Hypoxic treatment of primary AECs was shown to significantly increase (*p* < 0.001) the expression of the cytoplasmic pro-survival molecule Bcl-2. However, no significant difference was observed in the nuclear translocation compared to hypoxic control group (Figure 5B). Treatment with BMSC-CM significantly increased nuclear (*p* < 0.001) translocation of Bcl-2, compared to hypoxic control. Similarly, ADSC-CM significantly increased cytoplasmic expression (*p* < 0.001) and nuclear (*p* < 0.001) translocation of Bcl-2. Hypoxic treatment significantly increased cytoplasmic (*p* < 0.001) expression and nuclear translocation (*p* < 0.001) of the pro-apoptotic subunit, Bax (Figure 5C). Primary AECs treated with BMSC-CM showed significantly decreased cytoplasmic (*p* < 0.001) and increased nuclear translocation of Bax, compared to hypoxic control. ADSC-CM treatment also significantly decreased Bax in the cytoplasm (*p* < 0.001) and was more efficacious in significantly inhibiting its translocation to the nucleus, compared to hypoxic control (*p* < 0.05) and BMSC treatment (*p* < 0.001). The role of Bax in the cytoplasm and mitochondria is the activation of the apoptosis pathway. However, Bax exerts non-apoptotic functions when translocated into the nucleus. Interestingly, active caspase-3 was not detected in the cytoplasm of primary AECs in either hypoxic control and MSC-CM pre-treated groups (Figure 5D). Hypoxic treatment was shown to significantly enhance nuclear accumulation (*p* < 0.001) of the active caspase-3 subunit, while a significant reduction was observed in BMSC-CM (*p* < 0.001) and ADSCM (*p* < 0.01) treated cells suggesting MSC-CM protected against hypoxia-induced apoptosis through the inhibition of caspase-3. Primary AECs treated with hypoxia had significantly increased cytoplasmic phosphorylation (*p* < 0.001) and nuclear translocation (*p* < 0.001) of phosphorylated p38, compared to normoxic control (Figure 5E). This confirmed activation of oxidative stress pathways during hypoxic injury. Furthermore, treatment with both MSC-CM groups inhibited phosphorylation of p38 in the cytoplasm (*p* < 0.001). BMSC-CM further significantly increased (*p* < 0.001) the nuclear translocation of phosphorylated p38, while ADSC-CM significantly inhibited (*p* < 0.05) the nuclear accumulation of phosphorylated p38 compared to hypoxic control. This suggests that MSC-CM confer their modulatory effects in hypoxic injured primary AECs at least in part through the inhibition of the p38 signalling pathway.

### 2.6. Enhancement of Protective GRPs by MSC Secretome

**Figure 6:**
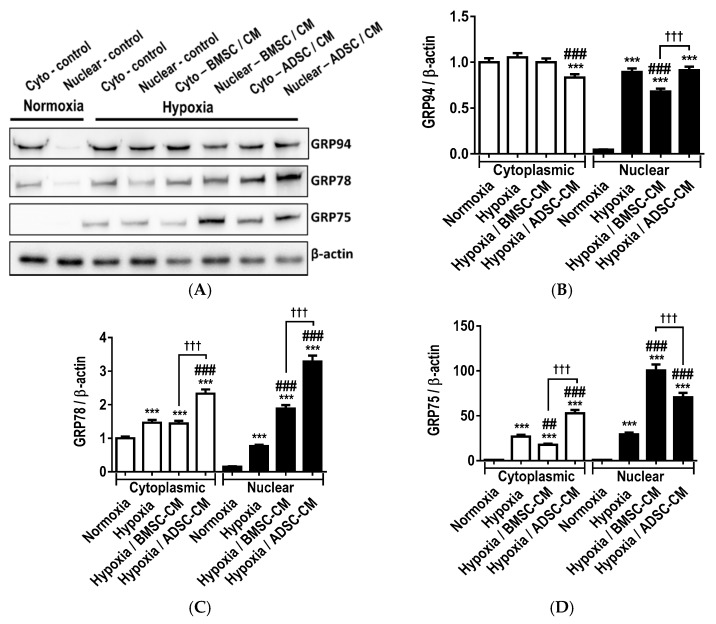
(**A**) Western blot analysis of modulatory effects of human MSC-CM on the expression of GRP intercellular chaperones in primary rat AECs exposed to hypoxia (1.5% O_2_) for 24 h. Cytoplasmic and nuclear accumulation of (**B**) GRP94, (**C**) GRP78 and (**D**) GRP 75 were quantitated by densitometry and normalized to β-actin. Data presented as mean  ±  SD; *n* = 3 (*** *p*  <  0.001 vs. normoxia control; ## *p*  <  0.01, ### *p*  <  0.001 vs. hypoxia control and ††† *p*  <  0.001 vs. hypoxia / BMSC-CM). The resulting data were statistically analysed using one-way ANOVA and Tukey’s multiple comparison test.

Western blot analysis (Figure 6A) of primary rat AECs pre-treated with MSC-CM exposed to hypoxic injury (1.5% O_2_) demonstrated modulatory effects on cytoplasmic expression and nuclear translocation of the endoplasmic reticulum residual chaperones, GRP94 and GRP78 and mitochondrial GRP75. Under normoxic physiological conditions, the basal expression of GRP94 (Figure 6B) and GRP78 (Figure 6C) in the cytoplasm was relatively abundant, with lower levels observed in the nucleus. Hypoxic treatment significantly increased the expression of GRP94 in the cytoplasm (*p* < 0.01) and nucleus (*p* < 0.001), compared to normoxic control. Treatment of primary AECs with BMSC-CM significantly lowered the expression of GRP94 in the cytoplasm (*p* < 0.01) and nucleus translocation (*p* < 0.001) compared to hypoxic control. There was no statistical significance observed in the cytoplasm expression and translocation of GRP94 in cells pre-treated with ADSC-CM. Hypoxic treatment significantly increased the expression of cytoplasmic (*p* < 0.001) and translocated nuclear (*p* < 0.001) GRP78. These effects were enhanced by BMSC-CM in the nucleus (*p* <0.001), while ADSC-CM showed further significant enhancement in both cytoplasmic (*p* < 0.001) and nuclear (*p* < 0.001) translocation, compared to BMSC-CM. Although no statistical significance was observed in the cytoplasmic expression or nuclear translocation of GRP75 (Figure 6D) in normoxia, hypoxic treatment significantly enhanced cytoplasmic levels (*p* < 0.001) and nuclear (*p* < 0.001) translocation of GRP75. Primary AECs treated with MSC-CM further enhanced the expression of GRP75 (*p* < 0.001), with BMSC-CM (*p* < 0.001) being the more efficacious of the MSC-CM groups at promoting nuclear translocation. Similar trends of significant modulation of cytoplasmic and nuclear translocation of GRP94, GRP78 and GRP75 by MSC-CM was also observed in A549 cells exposed to more severe hypoxia (0.5% O_2_) (Figure S3).

### 2.7. Protective and Immunomodulatory Effects of Recombinant GRPs

**Figure 7:**
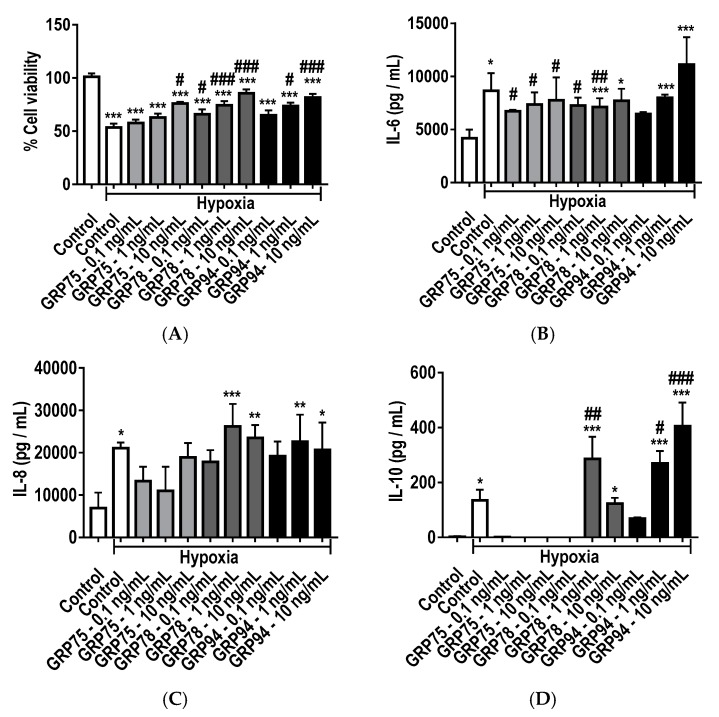
(**A**) Cell viability and release of pro-inflammatory (**B**) IL-6, (**C**) IL-8 and anti-inflammatory (**D**) IL-10 cytokines from A549 cells treated with recombinant glucose-regulated proteins (GRP75, GRP78 and GRP94) treatment during hypoxic (0.5% O_2_) exposure for 24 h. ELISAs were used to measure secreted cytokines in the supernatant of A549 cells. Data presented as mean  ±  SD; *n*  =  3 (* *p*  < 0.05, ** *p*  <  0.01, *** *p*  <  0.001 vs. normoxia control; and # *p*  <  0.05. ## *p*  <  0.01, ### *p*  <  0.001 vs. hypoxia control). The resulting data were statistically analysed using one-way ANOVA and Tukey’s multiple comparison test.

MTT assay (Figure 7A) revealed a significant increase in the viability of A549 cells pre-treated with increasing concentrations (0.1, 1 and 10 ng/mL) of recombinant GRP75, GRP78 and GRP98 and subjected to hypoxic exposure (0.5% O_2_). A significant increase in cell viability was observed with recombinant GRP75 at 10 ng/mL (*p* < 0.05); GRP78 at 0.1 ng/mL (*p* < 0.05); GRP78 at 1 ng/mL (*p* < 0.001); GRP78 at 10 ng/mL (*p* < 0.001); and GR94 at 1 ng/mL; and GRP94 at 10 ng/mL (*p* < 0.001) compared to hypoxia control. This demonstrated that increased concentrations of GRP could preserve cell viability and protect against hypoxic injury. ELISAs were used to measure the modulation of inflammatory cytokines IL-6, IL-8 and IL-10, produced by primary AECs treated with recombinant GRP chaperones. Treatment with hypoxia significantly (*p* < 0.05) increased the production of IL-1β (Figure 7B) compared to normoxic control, indicating enhanced inflammation. The presence of recombinant GRPs significantly reduced the release of IL-1β compared to hypoxia injury control, particularly with GRP75 at 0.1–10 ng/mL and GRP78 at 0.1–1 ng/mL, illustrating the immunomodulatory effects of GRPs. Likewise, the secretion of IL-8 (Figure 7C) by A549 cells treated with recombinant GRP78 at 1 ng/mL (*p* < 0.001) and 10 ng /mL (*p* < 0.01); and GRP94 at 1 ng/mL (*p* < 0.01)–10 ng/mL (*p* < 0.05) showed significant increase compared to normoxic control, however no significant difference was observed with the GRP treatment groups compared to hypoxia control. Nevertheless, the presence of recombinant GRP78 at 1 ng/mL (*p* < 0.01), GRP94 at 1 ng /mL (*p* < 0.05) and 10 ng/mL (*p* < 0.001) evoked a significant increase in anti-inflammatory cytokine IL-10 levels (Figure 7D) compared to normoxic control. These findings illustrate that there is an optimal GRP concentration for enhancement of anti-inflammatory effects.

### 2.8. Inhibition of GRPs Reduces the Protective Effects of MSC Secretome

**Figure 8:**
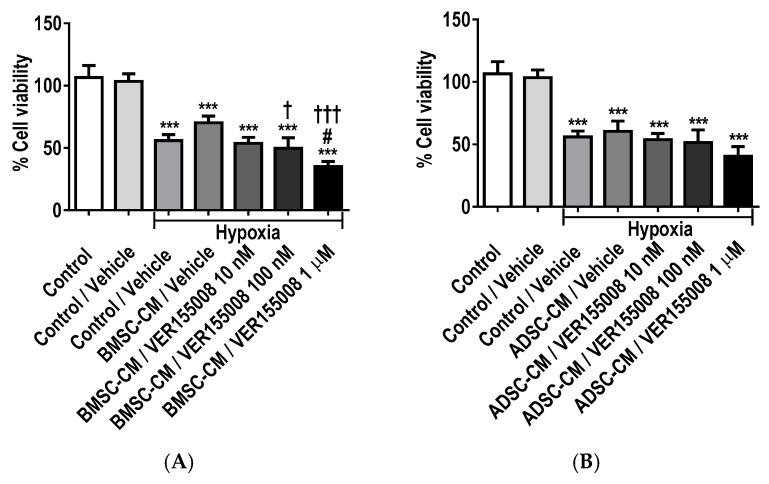

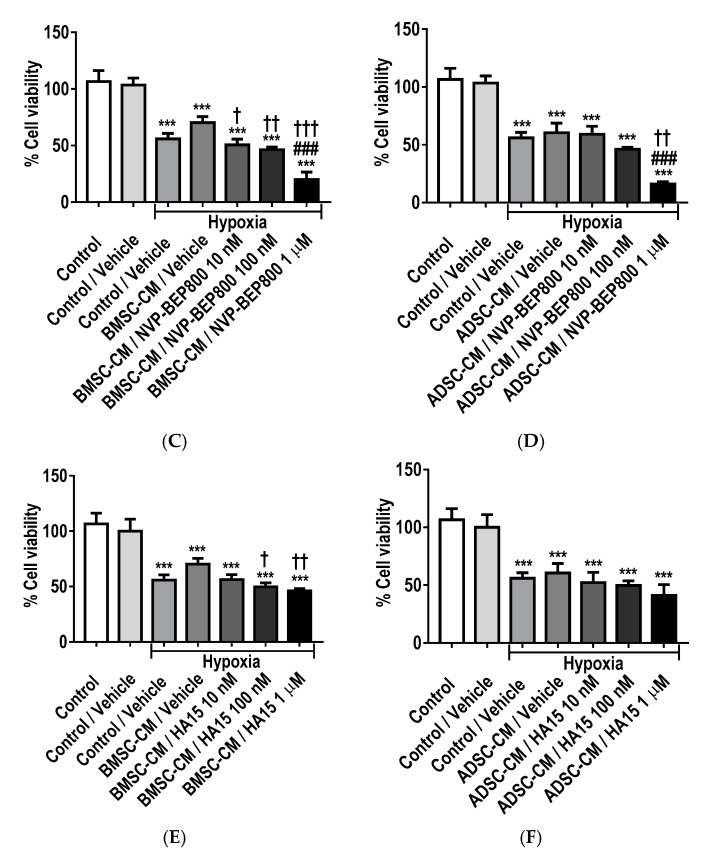
Cell viability of A549 cells treated with HSP70/90 family inhibitors, (**A**,**B**) VER155008 (HSP70 family; GRP75 and HSP70), (**C**,**D**) HA15 (GRP78) and (**E**,**F**) NVP-BEP800 (HSP90 family; GRP94, HSP90 and trap-1) in human MSC-CM during hypoxic (0.5% O_2_) exposure for 24 h. The viability of A549 cells was assessed via MTT assay. Data presented as mean  ±  SD; *n* = 3 (*** *p*  <  0.001 vs. normoxia control; and # *p*  <  0.05, ### *p*  <  0.001 vs. hypoxia control and † *p*  <  0.05, †† *p*  <  0.01, ††† *p*  <  0.001 vs. BMSC-CM / vehicle or ADSC-CM / vehicle). The resulting data were statistically analysed using one-way ANOVA and Tukey’s multiple comparison test.

MTT assay (Figure 8) showed a significant decrease in cell viability of A549 cells treated with HSP70/90 family inhibitors; VER155008 (HSP70 family; GRP75 and HSP70), HA15 (GRP78) and NVP-BEP800 (HSP90 family; GRP94, HSP90 and trap-1). A decrease in cell viability was observed with, increasing concentrations (10 nM, 100 nM and 1 µM) of VER155008, HA15 and NVP-BEP800 inhibitors in MSC-CM treated lung epithelial cells subjected to hypoxia (0.5% O_2_) for 24 h, compared to normoxic control. A further significant (*p* < 0.05) decrease in cell viability was exhibited in BMSC-CM with VER155008 inhibitor at 1 µM compared to hypoxic control vehicle and a further decrease was observed in BMSC-CM effects with VER155008 inhibitor group at 100 nM (*p* < 0.05) and 1 µM (*p* < 0.001) (Figure 8A,B). This demonstrated that increased concentrations of GRP75/HSP70 inhibitor reduced cell survival and protection against hypoxia-induced injury. Both BMSC-CM and ADSC-CM displayed a significant decrease in lung epithelial cell preservation with NVP-BEP800 inhibitor at 1 µM (*p* < 0.001) compared to hypoxic vehicle control (Figure 8C,D). A further decrease in cell viability was observed in the BMSC-CM group with NVP-BEP800 inhibitor at 10 nM (*p* < 0.05), 100 nM (*p* < 0.01), 1 µM (*p* < 0.001) and only the 1 µM (*p* < 0.01) group for ADSC-CM with NVP-BEP800 inhibitor. Taken together, this data suggests that GRP94/HSP90/trap-1 inhibition reduces GRP capabilities as a cryo-protectant. A further significant decrease cell viability was seen in BMSC-CM with HA15 inhibitor at 100 nM (*p* < 0.01) and 1 µM (*p* < 0.001) concentration (Figure 8E,F). The inhibition of GRP78 also illustrated the importance of GRP influence as an identified part of the MSC protective secretome. Notably, ADSC-CM with GRP inhibitors seemed to be less prone to further reduction in cell viability, as concentrations increased compared to BMSC-CM containing GRP inhibitor groups.

## 3. Discussion

MSC-based therapy in preclinical studies and clinical applications have been administered at ischemic and hypoxic injured sites [20,21]. As a result, optimization of preconditioning [22], conditioning [23] or activation [24] of MSCs is significant for developing clinical cell-based therapies for such injuries. This study assessed the cytoprotective, immunomodulatory, cellular homeostatic and anti-apoptotic capabilities of the MSC secretome in mitigating injury in pulmonary epithelial cells. We also compared the efficiency of MSC secretomes derived from two different tissue sources; bone marrow aspirate and adipose tissue. These experiments also evaluated the involvement and interplay of GRPs in the protective mechanism of MSC secretome. In the literature, in vitro modelling of lung IRI involves exposure of pulmonary epithelial or endothelial cells to an environment similar to that observed during the lung transplant and implantation phase. These in vitro models have shown that the duration of cold aerobic storage or hypoxic exposure can have deleterious effects on survival, cellular homeostasis and permeability. Although these in vitro models are isolated systems, they are also a suitable means to decipher cellular responses and modulation of signalling pathways that reflect changes that occur in vivo during IRI. In this study, the model employed a combination of hypoxia using lowered oxygen tension (0.5–1.5% O_2_) and ischemia via serum deprivation. Several optimization experiments were performed to establish a suitable oxygen tension and exposure time for the pulmonary epithelial cells. The 1.5%oxygen tension model produced a consistent viability change with a target 50%cell death in primary rat AECs, whereas transformed A549 cells were more resistant to hypoxia exposure, needing to be subjected to more severe oxygen tension (0.5%) to obtain similar results to primary AECs exposed to 1.5%oxygen tension. The established hypoxic-induced in vitro model of lung IRI was quite reliable and reproducible for observing cellular changes that simulate the hypoxic-ischemic tissue environment.

We observed that treatment with conditioned medium derived from BMSC or ADSC offered significant protection by delaying cells entering apoptosis, preserving cell viability and reducing LDH release in primary AECs and A549 cells. Furthermore, the present study also demonstrated that BMSC-CM and ADSC-CM could comparably attenuate secretion of pro-inflammatory CINC-1/CXCL-1, IL-1β and IL-6, while there was an enhancement seen in the production of TNF-α and anti-inflammatory cytokine IL-10 from primary AECs. We have demonstrated the direct immunomodulatory capacities of BMSC-CM and ADSC-CM in modulating the inflammatory response of primary AECs subjected to hypoxia-ischemia. Previous studies on rodent lung IRI have shown that MSC treatment down-regulates MIP-2, IL-1β and TNF-α expression levels while enhancing the up-regulation of anti-inflammatory IL-10 and prostaglandin E2 in bronchoalveolar lavage fluid [25]. Therefore, we have confirmed and expanded upon these finding in our own in vitro models.

This study indicates that pre-treatment with both MSC-CM significantly restores MMP physiological function in primary AECs subjected to hypoxic stress, thereby promoting repair and cellular homeostasis in injured cells. Injury index and cellular homeostasis of injured tissue are critically dependent on MMPs. During normal physiological conditions, the activity of these enzymes is low. However, during inflammation and reparative processes the activity of these enzymes is increased. Our established model produced a consistent injury index with an apparent impaired function of MMP-2 and -9 during hypoxic injury. MMP-2 and MMP-9 are responsible for the degradation of IL-1β into biologically inactive fragments through cleavage by MMP-2 at the Glu25-Leu26 bond [26]. This inactivation and reduced secretion are demonstrated in the presence of MSC-CM and may partially explain the anti-inflammatory effects seen. Conversely, IL-1β precursor molecule is known to be processed by MMP-2 and MMP-9 into biologically active forms [27]. Thus, the IL-1β activity can be both upregulated and downregulated by MMPs during acute or chronic inflammation. The function of MMPs is essential for the regulation of the inflammatory response to injury. These enzymes can control the bioavailability of several inflammatory mediators and growth factors through proteolytic cleavage from their membrane-anchored form. Similar to the case of IL-1β, previous studies have shown that MMP-2 and MMP-9 cleave membrane-bound TNF-α and thereby release a soluble and active precursor of the pro-inflammatory cytokine [28,29]. If MSC-CM-induced MMP function was indeed acting on the inflammatory response, these findings could potentially explain the increased TNF-α observed in our hypoxic injured primary AECs.

The Bcl-2 family is extensively studied with regards to their roles in the cytoplasm and mitochondria in relation to cell death [30]. They have been reported to translocate from the cytosol to the nucleus in response to various stress stimuli that induce apoptosis [31]. However, a recent study by Brayer, et al. [32] has demonstrated the non-apoptotic functions of Bax protein in the maintenance of cellular homeostasis and its role in lung tissue remodelling. They also showed that activation of nuclear Bax can influence cell growth and differentiation of A549 and primary human lung fibroblasts (HLF). Their finding illustrates the involvement and localization of Bax protein in the nucleus and its relative association with cell cycle progression and the chromatin compartment [32]. BMSC-CM demonstrated a significantly increased capability for translocating Bax protein from the cytoplasm into the nucleus, while ADSC-CM inhibited this effect, demonstrating their significantly different mechanisms of actions. If the nuclear localization of Bax confers recovery processes for cellular restoration to homeostasis, we can speculate that the cytoprotective effects of MSC-CM against hypoxia-induced injury may involve the nuclear accumulation of Bax. Previous studies have shown that caspase-3 can be translocated from the cytoplasm into the nucleus after induction of apoptosis, thereby enhancing the nuclear accumulation of active caspase-3 during apoptosis [33]. The present study has demonstrated that both MSC-CM was effective at reducing the accumulation of active caspase-3 in the nucleus, suggesting that the MSC-CM secretome inhibits the activity of effector caspase-3, thereby protecting against cell death.

Apoptotic cell death in response to inflammatory mediators has been associated with the p38 MAPK signalling complex, which is also involved in oxidative stress and inflammation in a variety of cell types. Activation of the MAPK signalling pathways has been shown to be one of the significant contributors to mediating the inflammatory response during lung IRI [34]. In several models of lung IRI, inhibition of p38 reduced the release of pro-inflammatory cytokines, alleviated alveolar injury and pulmonary oedema in addition to exerting anti-apoptotic properties [35,36]. The presented study demonstrated that pre-treatment with ADSC-CM significantly attenuated the activation of p38 in primary AECs exposed to hypoxia. In contrast, BMSC-CM further enhanced translocation and accumulation of phosphorylated p38 in the nucleus, which has previously been shown to be a reparative mechanism. Sakiyama, et al. [36] demonstrated the activation of p38-α to produce anti-apoptotic properties through the inhibition of caspase-3 in a rodent lung injury model. Cell heterogeneity can attribute such inconsistencies in the activation and effects of p38, secreted molecules and differential modulation of p38 isoforms (α, β, γ and δ), as each isoform has different effects on apoptotic activities [37]. This suggests that BMSC and ADSC secretome may have different soluble factors that could affect p38 activation. Both of the MSC-CM reduced hypoxia-induced injury of primary AECs, by mechanisms which may include inhibition of p38 phosphorylation and nuclear translocation. Pre-treatment with both MSC-CM enhanced translocation of Bcl-2 to the nucleus following hypoxic injury.

The intracellular molecular chaperones GRP75, GRP78 and GRP94, are localized in the mitochondrial and endoplasmic reticulum (ER), respectively [7]. GRPs are central regulators of cellular homeostasis, secretory pathways and the unfolded protein response (UPR) [38]. Induction of these chaperones during cellular stress is an adaptive and pro-survival measure [39,40] The GRPs are traditionally known to reside in the ER but accumulating evidence is emerging that isoforms of GRP78 can also be detected in other cellular locations, such as mitochondrial, cytoplasm, surface, nucleus as well as being secreted [41,42,43,44]. Therefore, protein function can be altered in response to changes in the environment, assuming novel functions that control signalling, proliferation, inflammation, apoptosis and oxidative stress [45]. Previous studies have reported a correlation between the level of GRP78/94 induced in cells and their resistance to apoptosis which reaffirms that GRPs possess anti-apoptotic properties [46,47,48]. According to Barker, et al. [49], the nuclear form of GRP78 has been shown to crosslink with DNA and may be involved in the suppression of DNA damage-induced apoptosis via a distinct nuclear regulatory mechanism of action for cell adaptation and survival. Thus, it would be quite valuable to have localization or translocation of GRP78, GRP94 and mitochondrial GRP75 to the nucleus for regulatory purposes. Also, a localized form of GRP78 in the nucleus may be crucial for cell viability and anti-apoptosis processes during cellular stresses [44]. Therefore, based on the current findings, we can only speculate that a subpopulation of these GRPs translocate into the nucleus and protect against hypoxic injury via a distinct regulatory mechanism. Induction secreted GRP78 can bind to the cell surface receptor and activate pro-survival kinase signalling. [44] However, it has also been observed that extracellular recombinant GRP78 does not bind to the cell surface, as a result of bound cell-surface GRP78 formally secreted by the cells [50]. Thus, recombinant GRPs introduced into the media may be inducing autocrine signalling through distinct cell surface receptors leading to altered transcription/expression that influences survival factors in stressful environments. Secreted GRPs initiate signal transduction pathways by binding to the surface receptor of adjacent cells. It has been reported that HSP70 uses both toll-like receptors (TLR) 2 and 4 synergistically to induce the production of IL-1β, TNF-α, IL-6 and IL-12 via a myeloid differentiation primary response 88 (MyD88)-dependent and cluster of differentiation (CD)14-dependent cascades [51,52]. Extracellular HSP70 has been shown to bind to the plasma membrane with high affinity, stimulating an instant intracellular calcium ion (Ca^2+^) flux and increasing the secretion of pro-inflammatory cytokines through the activation of nuclear factor kappa B (NF-κB), MyD88 and insulin receptor kinase (IRK) signalling in human monocytes [53]. Previous studies have demonstrated that extracellular receptors CD40 [54,55], CD91 [56] and c-c chemokine receptor type 5 (CCR5) [57] specifically bind and mediate the uptake of exogenous human HSP70, HSP90, GRP94 peptide complexes thereby eliciting cytoprotective effects. Moreover, GRP78 and GRP94 are potent anti-apoptotic proteins that also form complexes and sequester pro-apoptotic signalling molecules; procaspase-7 [58,59] and Bcl-2-interacting killer (BIK) [60].

The enhanced extracellular expression of intracellular HSPs and GRPs have emerged as significant mediators of transport and effector functions. Asea et al. [51] have recently termed the HSP and GRP family as “chaperokines” due to exhibiting dual role as chaperones and innate immunity mediators. These chaperones have also been identified as modulators of pro-inflammatory mediators when secreted from their residual complexes into the extracellular milieu [61]. It is well known that GRP molecular chaperones are intercellularly localized, nonetheless, Aksoy et al. [62] have previously shown that GRP78 is secreted constitutively by human AECs. The extracellular function of these chaperokines have been previously shown to be cytoprotective and cyto-stimulatory, including an ability to potently modulate inflammatory cytokine and chemokine production as well as upregulation of co-stimulatory factors [52]. Vega, et al. [63] reported that translocation of membrane-associated forms of these chaperones into the extracellular environment could act as a danger signal for the activation of the immune system during stress conditions. Evaluation of the functional responses mediated by GRPs is a significant step to establishing these chaperones as important mediators of the therapeutic effects observed with MSC therapy. In this context, we confirmed that pre-treatment with both MSC-CM enhanced cytoplasmic expression and nuclear translocation of GRPs in pulmonary epithelial cells injured by hypoxia. The study showed that ADSC-CM was significantly more effective at increasing the production of GRPs in lung epithelial cells. The presented work also demonstrated that recombinant GRPs confer protective and immunomodulatory effects in response to hypoxic injury. Pre-treatment with recombinant GRPs protected pulmonary epithelial cells in a dose-dependent manner, in addition to down-regulating inflammatory mediators. In contrast, pharmacological inhibition of the chaperone families was detrimental to cellular survival. Previous studies have shown that preconditioning MSC with recombinant HSP90 molecular chaperone can protect against hypoxia-induced apoptosis via phosphatidylinositol 3-kinase (PI3K), protein kinase B (Akt) and extracellular signal-regulated protein kinases (ERK1/2) pathways and enhancing paracrine activity [6]. A study by Chase, et al. [5], showed that recombinant HSP72, induced pro-inflammatory cytokines through the TLR4/NF-κB signalling. Other previous studies have shown that GRPs are negative regulators of inflammatory processes. GRP78 has been shown to inhibit the production of pro-inflammatory cytokines (TNF-α, IL-6 and IFN-β) induced by lipopolysaccharide (LPS) in bone marrow-derived dendritic cells (BMDCs) and bone-marrow-derived macrophages (BMDMs) [64]. Additionally, transient knockdown of GRP78 has been shown to abolish the secretion of serum ferritin mediated pro-inflammatory cytokines (IL-1β, IL-6 and TNF-α) in rat primary activated hepatic stellate cells [65]. If GRPs are essential chaperones for the protection of cells, their elimination could result in detrimental effects on various biological functions. Our findings strongly indicate GRPs stimulate endogenous protective mechanisms and function as modulators of inflammation, which makes these proteins a promising base for novel clinical therapies.

## 4. Materials and Methods

### 4.1. Material

All tissue cultures plastics and reagents were purchased from Sarstedt (Drinagh, Ireland) and Sigma Aldrich (Arklow, Ireland) unless otherwise stated.

### 4.2. Cell Culture

Transformed human type II alveolar epithelial cells (A549, American Type Culture Collection, Manassas, VA, USA) were cultured in high glucose Dulbecco’s Modified Eagle’s Medium (DMEM) supplemented with 10% foetal bovine serum (FBS) and 10,000 U/mL penicillin (Fisher Scientific, Dublin, Ireland), 10,000 μg/mL streptomycin (Fisher Scientific, Dublin, Ireland).

### 4.3. Conditioned Medium

Commercially available human bone marrow mesenchymal stem cells (BMSC, Lonza, Basel, Switzerland) and human adipose-derived mesenchymal stem cells (ADSC, Lonza, Basel, Switzerland) were cultured at an initial density of 5 × 10^5^ cells in T175 flasks with minimum essential medium-α (MEM-α) with glutamate (Fisher Scientific, Dublin, Ireland), supplemented with 10% FBS, 1 ng/mL basic fibroblast growth factor (bFGF) and 10,000 U/mL penicillin (Fisher Scientific, Dublin, Ireland), 10,000 μg/mL streptomycin (Fisher Scientific, Dublin, Ireland). Conditioned media was prepared from 80% to 90% confluent MSC cultures between passages 2 and 4. Confluent cells were cultured in serum-free MEM-α supplemented with penicillin-streptomycin then incubated under normoxic (21% O_2_, 5% CO_2_) conditions for 24 h. The subsequent conditioned medium was collected, 0.45 μm filtered to remove cell debris and aliquoted and stored at −20 °C.

### 4.4. Primary Cell Isolation and Culture

Tissue harvesting for primary type II AEC isolation was performed with the approval of the Animal Care Research Ethics Committee (ACREC) of National University of Ireland Galway. Primary AECs from pathogen-free male Sprague-Dawley (200–250 g) were isolated as described previously [66]. Briefly, subsequent to euthanasia, perfusion and lavage of lungs was performed via a tracheal cannula, both trachea and lungs were removed from the thoracic cavity. Lung tissue was digested with elastase solution for 15 min at 37 °C, chopped in 5 mL FBS and 10 mL DNase solution (10 mg/mL phosphate buffer saline (PBS), Fisher Scientific, Ireland) for 10 min and then shaken for 5 min. The digest was filtered through two nylon filters (40 μm and 100 μm, respectively). The filtered lung solution was centrifuged (110 g, 8 min, 4 °C); the pellet was resuspended in un-supplemented DMEM and incubated (21% O_2_, 5% CO_2_) at 37 °C for 45 min in a Petri dish to allow for the attachment of macrophages. The unattached cells were then centrifuged (110 g, 8 min, 4 °C) and resuspended in the final DMEM medium containing; 100 mM sodium pyruvate, 2 mM L-glutamine, 10% FBS, 10,000 U/mL penicillin, 10,000 μg/mL streptomycin and 250 mU/mL amphotericin-β. The cells were counted with a haemocytometer and plated on 6-well plates (4 × 10^5^ cells/well). The cells were used 72 h following plating after they had been rinsed with un-supplemented DMEM to remove non-adherent cells.

### 4.5. Exposure to Hypoxia

For hypoxia treatment, confluent monolayers were pre-treated with fresh medium or MSC-CM and immediately subjected to hypoxia at a desired O_2_ tension, as previously described [67]. Briefly, primary rat AECs and A549 cells were plated and cultured until 80–90% confluent, the cell monolayers were then pre-treated with BMSC, or ADSC conditioned medium prior to being placed in a humidified airtight hypoxic chamber (COY Laboratory Products, Grass Lake, MI, USA) with automated sensor controllers for O_2_, CO_2_, humidity and temperature. Cells were exposed to constant O_2_ levels, primary rat AECs (1.5% O_2_, 5% CO_2_) and A549 cells (0.5% O_2_, 5% CO_2_) and were maintained at 37 °C for 24 h. Hypoxia exposure was followed by 1 h of reoxygenation in normoxia (21% O_2_, 5% CO_2_). Control cells were exposed to normoxic conditions and were also maintained at 37 °C for the same duration of 24 h. Subsequent to hypoxic/normoxic culture, samples were further assessed or collected and frozen at −20 °C.

### 4.6. Flow Cytometric Analysis of Apoptosis

Detection of apoptosis was performed by flow cytometry using an Annexin V-fluorescein isothiocyanate (FITC)/Propidium Iodide (PI) apoptosis detection kit (BD Bioscience, Oxford, UK), as previously described [68]. Briefly, primary AECs were seeded in 6-well plates and treated with BMSC-CM or ADSC-CM and then subjected to hypoxia (1.5% O_2_) for 24 h. The treated cells were harvested and washed twice with ice-cold PBS and centrifuged. The cell pellets were resuspended in 1× annexin-binding buffer and incubated on ice, followed by the addition of Annexin V-FITC/PI according to the manufacturer’s instructions. The stained cells were then analysed by flow cytometry (BD Accuri™ C6, BD Biosciences, Oxford, UK) and analysed using BD Accuri™ C6 analysis software version 1.0264.21.

### 4.7. Cell Viability and Proliferation Assay

MTT (3-(4, 5-dimethylthiazol-2-yl)-2,5-diphenyltetrazolium bromide) assay was performed using MTT reagent Thiazolyl Blue Tetrazolium Bromide reconstituted in culture medium (5 mg/mL) to evaluate cell viability and proliferation, as has been described previously [69]. Briefly, cells were seeded at 100,000 cell/cm^2^ on 96-well culture plates and exposed to treatment conditions: BMSC-CM or ADSC-CM, recombinant proteins; HSPA5, HSPA9 or HSP90β1 (Aviva Systems Biology, San Diego, CA, USA) and HSP 70/90 family inhibitors; VER155008, HA15 or NVP-BEP800 (Selleckchem, Munich, Germany). After treatment, cells were washed with PBS, followed by incubation with MTT solvent for 3 h at 37 °C in a humidified incubator. Cell supernatant was replaced with dimethyl sulfoxide (DMSO) and absorbance readings were measured using the Varioskan™ Flash microplate reader (Thermo Scientific, Loughborough, UK) at 595 nm wavelength. The degree of cell viability was presented as a percentage of optical density (OD) relative to control.

### 4.8. Lactate Dehydrogenase (LDH) Assay

LDH release was assessed by CytoTox 96^®^ LDH assay (Promega, Southampton, UK) as previously described [69], as per manufacturer’s protocol. Briefly, the cell supernatant was mixed with supplied assay buffer and absorbance readings were obtained at 490 nm wavelength using the Varioskan™ Flash microplate reader (Thermo Scientific, Loughborough, UK). The LDH release was presented as fold change compared to control absorbance.

### 4.9. Gelatin Zymography

The activity of MMPs secreted by cells was assessed by gelatin zymography as previously described [26,27]. Briefly, cell supernatant subsequent to treatments was collected and ran on 10% sodium dodecyl sulphate-polyacrylamide gel electrophoresis (SDS-PAGE) with 1 mg/mL gelatin. After the 90-min run at 120 V, the gels were then incubated in 2.5%Triton X-100 for 1 h to remove SDS. Followed by a 24 h incubation at 37 °C in 2.5% Triton X-100 solution with 5 mM calcium chloride (CaCl_2_), 1 μM zinc chloride (ZnCl_2_) and 50 mM Tris at pH 7.4. Subsequently, the gels were briefly rinsed in deionized H_2_O and stained for 30 min with Coomassie Brilliant Blue and imaged using multipurpose imaging system (UVITEC Imaging System, Cambridge, UK).

### 4.10. Western Blot Analysis

Immunoblots for protein identification and assessment were performed as previously described [70]. After treatment, total protein from cells was extracted using protease and phosphatase inhibitor cocktail supplemented radio-immunoprecipitation assay (RIPA) lysis buffer, followed by SDS-PAGE and transfer onto nitrocellulose membranes (GE Healthcare, Chicago, IL, USA). The membranes were blocked in Tris-buffered saline with 0.1% Tween 20 (TBS-T, Fisher Scientific, Dublin, Ireland) containing 5% bovine serum albumin (BSA) for 1 h, primary antibodies against rabbit anti-ACTIVE^®^-caspase-3 (1:250; Promega, Southampton, UK), mouse anti-Bcl-2 (1:1000, Santa Crus Biotechnology, Heidelberg, Germany), mouse anti-Bax and mouse anti-total-p38 (1:500 Santa Cruz Biotechnology, Heidelberg, Germany), rabbit anti-phospho-p38 (1:1000, Novus Bio-techne, Abingdon, UK), mouse anti-GRP75, mouse anti-GRP78 and rat anti-GRP94 and mouse anti-β-actin (1:1000, Santa Cruz Biotechnology, Heidelberg, Germany) were diluted in TBS-T with 5% skimmed milk overnight at 4 °C. After 24 h, the membranes were washed in TBS-T and incubated in the appropriate peroxidase-conjugated secondary antibody (1:1000; HuaAn Biotechnology, Hangzhou, China) for 1 h at room temperature. Subsequent to incubation with a luminescent substrate, signals from immune-reactive bands were visualized using a luminescence imaging system (UVITEC Imaging System, Cambridge, UK).

### 4.11. Enzyme-Linked Immunosorbent Assay (ELISA)

Cytokine levels were measured in the culture supernatants using standard ELISA kits as has been described previously [25]. Rat CINC-1/CXCL-1, IL-1β and TNF-α, rat and human IL-6 and IL-10 and human IL-8 were quantified using DuoSet^®^ ELISA kits (R&D Systems, Abingdon, UK). Briefly, polyester ELISA plates were pre-coated overnight with capture antibody against antigens, incubated and blocked in 100 µL PBS containing 1% BSA. ELISA wells were washed 3 times between every interval with 200 μL PBS-Tween. Biotin-conjugated secondary antibodies against the specific antigen were added to the plates and HRP-streptavidin after incubation. Detection was performed using 100 µL 3,3′,5,5′-Tetramethylbenzidine (TMB) chromogenic substrate, the reaction was terminated by adding 50 μL 1 N sulfuric acid (H_2_SO_4_) and the results were read at 450 nm with 550 nm background correction wavelengths using Varioskan™ Flash microplate reader (Thermo Scientific, Loughborough, UK).

### 4.12. Statistical Analysis

All data was accumulated repeatedly in three independent experiments/biological replicates (*n* = 3) and corresponding technical replicates. Data were analysed using statistical software (GraphPad Prism^®^ software version 7.0, La Jolla, CA, USA) and results were reported as a mean ± standard deviation and *p*-values less than 0.05 (*p* < 0.05) were considered significant. For single data sets One-way analysis of variance ANOVA was used to determine the significant differences between the experimental groups followed by Tukey’s multiple comparison post-hoc test.

## 5. Conclusions

In conclusion, we show here that MSC secretome is protective in hypoxic injured pulmonary epithelial cells, by delaying cells entering apoptosis and preserving cell viability and membrane integrity. Both MSC secretomes from different tissue sources exhibited immunomodulatory properties by changing the expression of inflammatory cytokines and restoring MMP activity during injury. MSC secretome was useful at evoking expression of GRPs and anti-apoptotic proteins while inhibiting the activation of p38 MAPK. We have shown that pulmonary epithelial cells respond to stressful environments by producing GRPs thus attenuating hypoxic injury, also proven through inhibition of inflammatory cytokine production at lower doses of recombinant GRP treatment, while higher doses activated the inflammatory response. We have demonstrated that inhibition of these molecular chaperones is detrimental to cell survival, proving that these chaperones play a crucial role in the cytoprotective mechanism of MSC secretome acting as a complementary mediator of cellular responses. This study indicates that MSC secretome could be a feasible approach for the induction of biologically active GRPs for the treatment of lung IRI.

## Supplementary Materials

Supplementary materials can be found at http://www.mdpi.com/1422-0067/19/10/2996/s1.

## Abbreviations

A549	Transformed type II alveolar epithelial cell
AEC	Alveolar epithelial cell
ADSC	Adipose-derived stem cell
Bax	Bcl-2-associated X protein
Bcl-2	B-cell lymphoma 2
BMSC	Bone marrow-derived mesenchymal stem cell
CM	Conditioned medium
CINC-1	Cytokine-induced neutrophil chemoattractant 1
CXCL-1	Chemokine (C-X-C motif) ligand 1
GRP	Glucose-regulated protein
IL	Interleukin
IRI	Ischemia-reperfusion injury
MAPK p38	Mitogen-activated protein kinase
MMP	Matrix metalloproteinases
MSC	Mesenchymal stem cell
TNF-α	Tumour necrosis factor alpha
