# Supplementary material for: Human Mesenchymal Stem Cell Secretome from Bone Marrow or Adipose-Derived Tissue Sources for Treatment of Hypoxia-Induced Pulmonary Epithelial Injury
Source: Int J Mol Sci. 2018 Sep 30;19(10):2996. doi: 10.3390/ijms19102996 (PMC6212866; doi:10.3390/ijms19102996)
Supplement: Supplementary file 1 [file ijms-19-02996-s001.pdf]

International Journal of *Molecular Sciences*

MDPI

Article

Supplementary Figures

MTT analysis (Figure S1A) of A549 cells pre-treated with MSC-CM and exposed to hypoxic injury (0.5 % O_2) showed increased cell preservation, though no significant differences in cell viability were observed MSC-CM pretreatment groups compared to hypoxic control. There was a significant increase of LDH release ($p < 0.001$) in A549 cells (Figure S1B) exposed to hypoxia, and pre-treatment with both MSC-CM significantly ($p < 0.01$) preserved cell membrane integrity reducing the release of LDH, compared to hypoxic control.

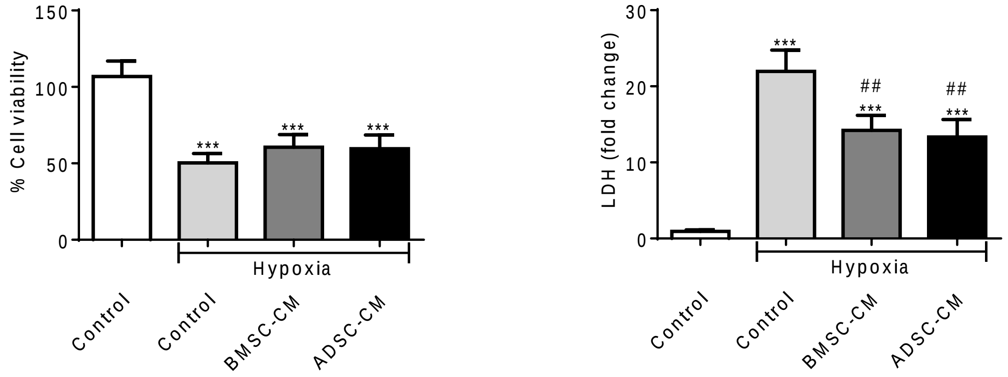

(**A**) (**B**)

Figure S1: (**A**) Cell viability and (**B**) LDH release of A549 cells treated with human MSC-CM during hypoxic (0.5% O_2) exposure for 24 h. The viability and LDH release of A549 cells were measured via MTT and LDH assays, respectively. Data presented as mean ± SD; n = 3 (***$p < 0.001$ vs normoxia control, and ##$p < 0.01$ vs hypoxia control). The resulting data was statistically analysed using one-way ANOVA and Tukey's multiple comparison test.

Western blot analysis (Figure S2A) of A549 cells pre-treated with MSC-CM and subjected to hypoxia (0.5 % O_2) exposure. Modulation of protein expression and activation of Bcl-2, Bax active caspase-3 and phosphorylated p38 were assessed to confirm anti-apoptotic, cytoprotective and induction of cellular stress signaling pathways. There was no significant difference observed in cytoplasmic and nuclear translocation of Bcl-2 between normoxic and hypoxic control group (Figure S2B), however, there was a significant increase in nuclear translocation ($p < 0.001$) in BMSC treated cells, thereby demonstrating an activation of this pro-survival molecule. Although, A549 cells treated with ADSC-CM showed significantly ($p < 0.001$) increased cytoplasmic and nuclear Bcl-2 compared to hypoxic control. BMSC-CM treatment showed further significant enhancement of nuclear translocated Bcl-2. Hypoxic treatment of A549 cells has been shown to enhance cytoplasmic protein expression ($p < 0.001$) and nuclear translocation ($p < 0.001$) of Bax, suggesting an activation of apoptotic signaling through this pro-apoptotic factor (Figure S2C). Although treatment with BMSC-CM further significantly enhanced the nuclear translocation ($p < 0.001$) of Bax, ADSC-CM treatment inhibited nuclear translocation ($p <0.001$) of Bax, demonstrating a pro-survival activation in the cells. A549 cells treated with hypoxia had significantly increased cytoplasmic expression (p <

0.01) and nuclear translocation (p < 0.01) of phosphorylated p38 compared to normoxic control, demonstrating activation of the oxidative stress pathways during hypoxic injury (Figure S2D). Furthermore, treatment with both MSC-CM groups enhanced this expression of phosphorylated p38 at cytoplasmic (p < 0.001) and nuclear levels (p < 0.001), while a further significant expression of cytoplasmic (p < 0.001) and nucleic (p < 0.001) phosphorylated p38 is observed with ADSC-CM treatment. This suggests that MSC-CM confer their modulatory effects in hypoxic injured A549 through the activation of p38 signaling pathway.

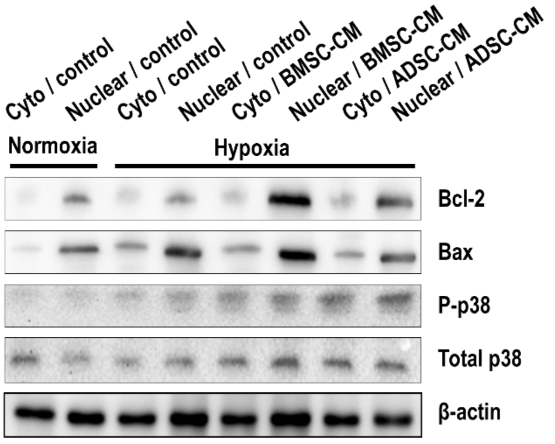

(**A**)

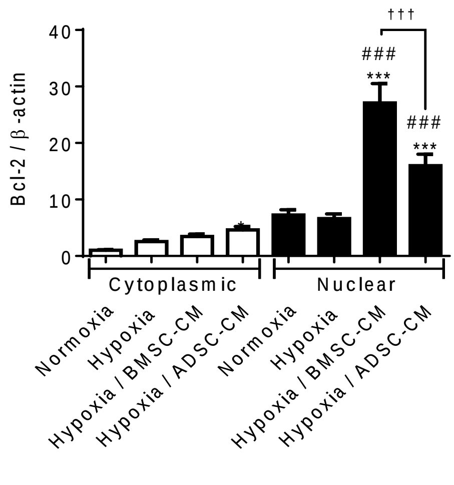

(**B**)

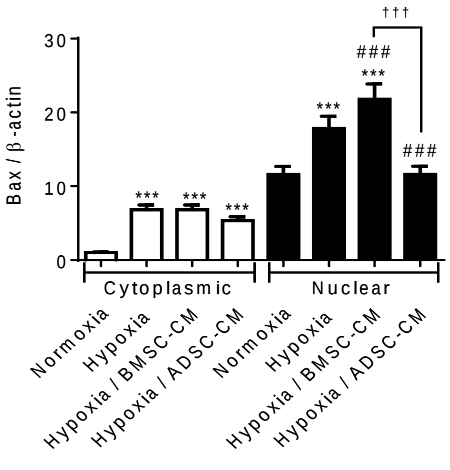

(**C**)

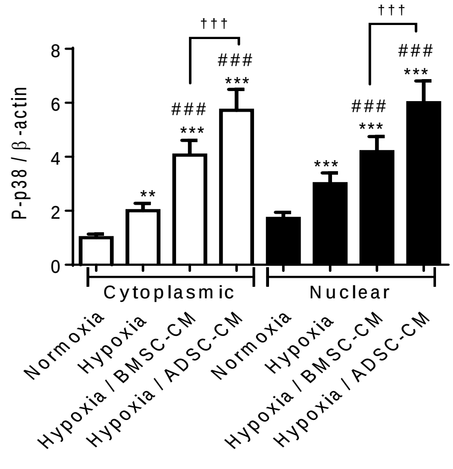

(**D**)

Figure S2: (**A**) Western blot analysis exploring modulatory effects of human MSC-CM on the activation of cellular stress signalling mediators in A549 cells exposed to hypoxia (0.5% O_2) for 24 h Cytoplasmic and nuclear accumulation of (**B**) Bcl-2, (**C**) Bax, and (**D**) phosphorylated p38 were quantitated by densitometry and normalized to β-actin. Data presented as mean ± SD; n = 3 (*p < 0.05, ** p < 0.01, *** p < 0.001 vs. normoxia control; ### p < 0.001 vs. hypoxia control and ††† p < 0.001 vs. hypoxia / BMSC-CM). The resulting data was statistically analysed using one-way ANOVA and Tukey's multiple comparison test.

Western blot analysis (Figure S3A) of hypoxic (0.5% O_2) injured A549 pre-treated with MSC-CM showed induction of cytoplasmic expression and nuclear translocation of ER residential chaperones, GR94 and GRP78, and mitochondrial GRP75. Induction of these chaperones during injury is a pro-survival mechanism. Under normoxic physiological condition, the basal expression of GRP in the cytoplasmic is relatively abundant, with lowered levels in the nucleus. Although there was no significant difference observed in cytoplasmic expression and nuclear translocation of GRP94 (Figure S3B), GRP78 (Figure S3C) and GRP75 (Figure S3D) between normoxic and hypoxic control groups, hypoxic exposed significantly reduced the expression of GRP75 in the nucleus ($p <0.001$), control to normoxic control. Treatment with BMSC-CM significantly increased the expression of cytoplasmic ($p < 0.05$) GRP94, compared to control, while ADSC-CM treatment further enhanced both cytoplasmic ($p < 0.001$) and nuclear ($p < 0.001$) as compared to BMSC-CM treatment group. Treatment with BMSC-CM enhanced the expression of cytoplasmic ($p < 0.001$) and nucleic ($p < 0.001$) GRP78, compared to hypoxic control, while ADSC-CM further significantly increased cytoplasmic phosphorylation ($p < 0.01$) and nucleic accumulation ($p < 0.001$). Lastly, A549 cells treated with BMSC-CM showed significant increases in cytoplasmic ($p < 0.05$), and significantly increased nuclear ($p < 0.05$) expression, compared to hypoxic control. Although treatment with BMSC-CM was more efficacious in the enhancement of cytoplasmic ($p < 0.05$) GRP7, compared to ADSC-CM, nuclear translocation ($p < 0.001$) was further significantly enhanced by ADSC-CM treatment.

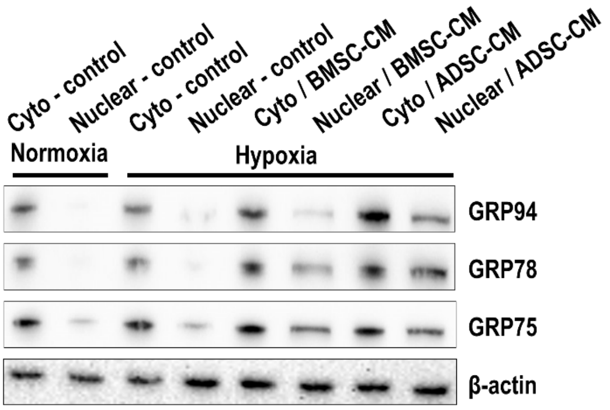

(A)

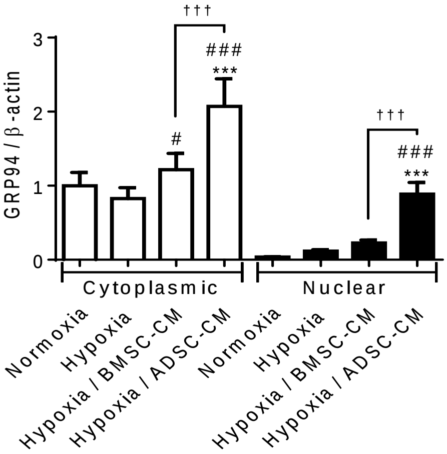

(B)

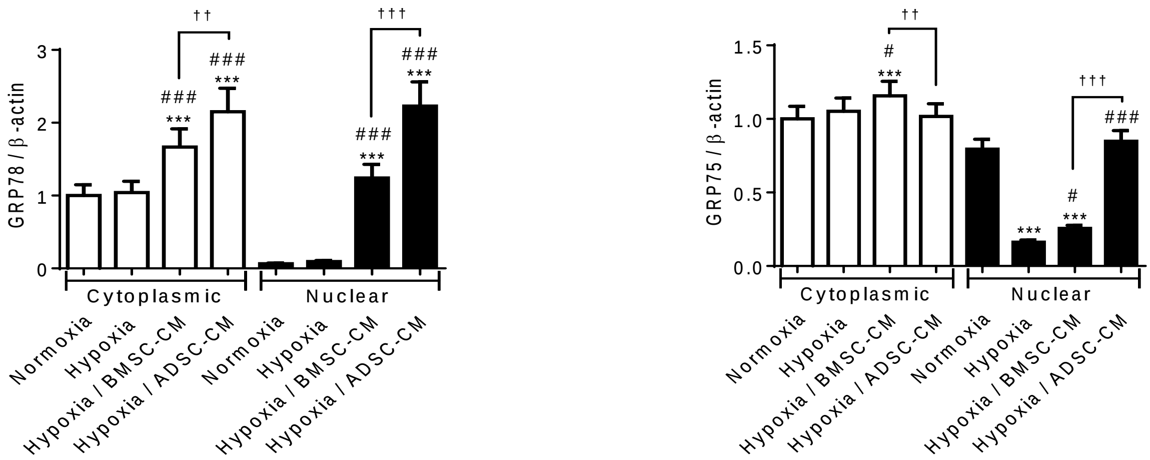

(**C**) (**D**)

Figure S3: (**A**) Western blot analysis of modulatory effects of human MSC-CM on the expression of GRP intercellular chaperones in A549 cells exposed to hypoxia (0.5% O_2) for 24 h. Cytoplasmic and nuclear accumulation of (**B**) GRP94, (**C**) GRP78 and (**D**) GRP 75 were quantitated by densitometry and normalized to β-actin. Data presented as mean ± SD; n = 3 (*** $p < 0.001$ vs. normoxia control; # $p < 0.05$, ### $p < 0.001$ vs. hypoxia control and †† $p < 0.01$, ††† $p < 0.001$ vs. hypoxia / BMSC-CM). The resulting data was statistically analysed using one-way ANOVA and Tukey's multiple comparison test.

© 2018 by the authors. Submitted for possible open access publication under the terms and conditions of the Creative Commons Attribution (CC BY) license (http://creativecommons.org/licenses/by/4.0/).
